# A predictive model using risk factor categories for hospital-acquired pneumonia in patients with aneurysmal subarachnoid hemorrhage

**DOI:** 10.3389/fneur.2022.1034313

**Published:** 2022-12-06

**Authors:** Sheng-Qi Hu, Jian-Nan Hu, Ru-Dong Chen, Jia-Sheng Yu

**Affiliations:** ^1^Department of Neurosurgery, Tongji Hospital, Tongji Medical College, Huazhong University of Science and Technology, Wuhan, Hubei, China; ^2^Department of Respiratory and Critical Care Medicine, Key Laboratory of Pulmonary Diseases of Health Ministry, Tongji Hospital, Tongji Medical College, Huazhong University of Science and Technology, Wuhan, China

**Keywords:** aneurysmal subarachnoid hemorrhage, hospital-acquired pneumonia (HAP), predictive model, risk factor, age, Glasgow coma scale (GCS), blood glucose, red blood cell width distribution standard deviation

## Abstract

**Objectives:**

To identify risk factors for hospital-acquired pneumonia (HAP) in patients with aneurysmal subarachnoid hemorrhage (aSAH) and establish a predictive model to aid evaluation.

**Methods:**

The cohorts of 253 aSAH patients were divided into the HAP group (*n* = 64) and the non-HAP group (*n* = 189). Univariate and multivariate logistic regression were performed to identify risk factors. A logistic model (Model-Logit) was established based on the independent risk factors. We used risk factor categories to develop a model (Model-Cat). Receiver operating characteristic curves were generated to determine the cutoff values. Areas under the curves (AUCs) were calculated to assess the accuracy of models and single factors. The Delong test was performed to compare the AUCs.

**Results:**

The multivariate logistic analysis showed that the age [*p* = 0.012, odds ratio (OR) = 1.059, confidence interval (CI) = 1.013–1.107], blood glucose (BG; >7.22 mmol/L; *p* = 0.011, OR = 2.781, CI = 1.263–6.119), red blood distribution width standard deviation (RDW-SD; *p* = 0.024, OR = 1.118, CI = 1.015–1.231), and Glasgow coma scale (GCS; *p* < 0.001, OR = 0.710, CI = 0.633–0.798) were independent risk factors. The Model-Logit was as follows: Logit(*P*) = −5.467 + 0.057 ^*^ Age + 1.023 ^*^ BG (>7.22 mmol/L, yes = 1, no = 0) + 0.111 ^*^ RDW-SD−0.342 ^*^ GCS. The AUCs values of the Model-Logit, GCS, age, BG (>7.22 mmol/L), and RDW-SD were 0.865, 0.819, 0.634, 0.698, and 0.625, respectively. For clinical use, the Model-Cat was established. In the Model-Cat, the AUCs for GCS, age, BG, and RDW-SD were 0.850, 0.760, 0.700, 0.641, and 0.564, respectively. The AUCs of the Model-Logit were insignificantly higher than the Model-Cat (Delong test, *p* = 0.157). The total points from −3 to 4 and 5 to 14 were classified as low- and high-risk levels, respectively.

**Conclusions:**

Age, BG (> 7.22 mmol/L), GCS, and RDW-SD were independent risk factors for HAP in aSAH patients. The Model-Cat was convenient for practical evaluation. The aSAH patients with total points from 5 to 14 had a high risk for HAP, suggesting the need for more attention during treatment.

## Introduction

Aneurysmal subarachnoid hemorrhage (aSAH) is a neurologic emergency associated with a 32%−67% mortality and many severe complications (1, 2). About one-third of aSAH patients suffer from systemic infections (predominantly pneumonia) that can contribute to excess mortality after SAH (2, 3). Hospital-acquired pneumonia (HAP) is a common complication associated with poor outcomes (4–6). Even though numerous studies focused on stroke-associated pneumonia and acquired clinically predictive scales (7–9), different types of the stroke influenced the occurrence of HAP and reduced the prediction accuracy (10). To further specifically evaluate the HAP factors in aSAH patients and create strategies to prevent HAP and improve outcomes, it is critical to identify and mitigate risk factors in aSAH patients (4, 11).

Studies focusing on HAP in patients with aSAH returned inconsistent results. Chen et al. (11) demonstrated the significance of the neutrophil-to-lymphocyte ratio (NLR) in 711 aSAH patients. Wang et al. found that risk factors for pneumonia in aSAH patients included advanced age, male sex, weekend admission, the World Federal Neurological Society (WFNS) grade, extensive enteral nutrition, endovascular treatment, and specific laboratory parameters (4). Some studies only tested their limited cohorts' accuracy and thresholds of associated risk factors (12, 13). Nevertheless, clinical work requires a valid and convenient model to predict HAP in aSAH patients. Therefore, we explored the risk factors for HAP in patients with aSAH and established a predictive model.

## Methods

### Patients

**Figure 1:**
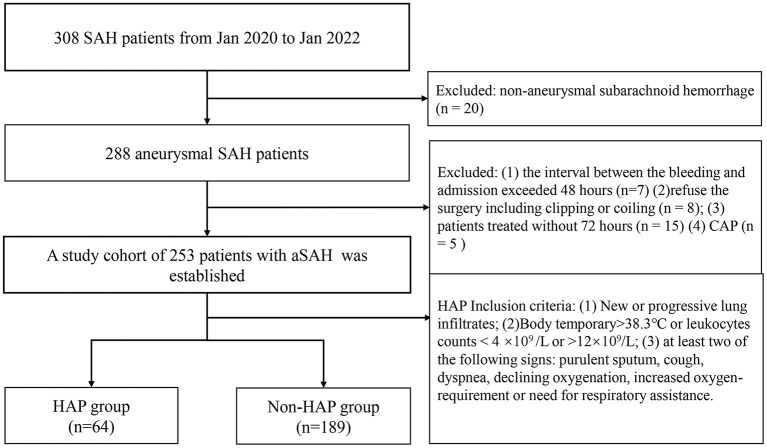
The flow chart of patient selection. SAH, subarachnoid hemorrhage; CAP, community-acquired pneumonia; HAP, hospital-acquired pneumonia.

The flow chart of patient selection is shown in Figure 1. Our hospital's institutional ethics committee approved this study. We collected data after obtaining the consent of the patients or their close relatives. From January 2020 to January 2022, 308 patients were diagnosed with SAH identified by computed tomography. There were 288 patients with a definitive diagnosis of an intracranial aneurysm according to computerized tomography angiography, digital subtraction angiography, or surgery at our institute (20 patients were excluded for non-aneurysmal hemorrhages). Exclusion criteria were as follows: (1) the interval between the bleeding and admission exceeded 48 h (*n* = 7); (2) the patient refused surgery, including clipping or coiling (*n* = 8); (2) the patients were treated without 72 h (*n* = 15); (4) there was a diagnosis of community-acquired pneumonia (*n* = 5). A cohort of 253 patients with aSAH was established and divided into a HAP group (*n* = 64) and a non-HAP group (*n* = 189). The HAP group's inclusion criteria were as similar to the literature (14), which was as follows: (1) new or progressive and persistent infiltrates; (2) body temperature of >38.3°C or leukocytes counts <4 × 10^9^/L or >12 × 10^9^/L; (3) at least two of the following signs: purulent sputum, cough, dyspnea, declining oxygen saturation, increased oxygen requirement, or need for respiratory assistance.

### Data

Clinical characteristics and laboratory tests are displayed in Table 1. The variables were as follows: (1) demographics; (2) comorbidities (i.e., hypertension, diabetes mellitus, hyperlipidemia), and history of smoking and drinking; (3) clinical scores [Glasgow coma scale (GCS), Hunt-Hess score, WFNS; modified Fisher grade]; (4) aneurysm location and occurrence of intracerebral hemorrhage and intraventricular hemorrhage; (5) choice of treatment including coiling or clipping; (6) laboratory tests (peripheral venous blood samples): blood glucose (BG); red blood cell (RBC); hematocrit; hemoglobin, platelets, white blood cell (WBC), neutrophils; monocytes, NLR, platelet to lymphocyte ratio, lymphocyte-to-monocyte ratio; platelet-to-WBC ratio, systemic inflammation response index (SIRI), red blood cell distribution coefficient of variation and red blood cell distribution width-standard deviation (RDW-SD). All clinical characteristics and laboratory tests were collected within 12 h of admission.

**Table 1:** Clinical characteristics and univariate analysis of the cohorts.

**Variables**	**HAP group** **(*n* = 64)**	**Contrast group** **(*n* = 189)**	***p-*value**	**Threshold**
Age	59.59 ± 7.84	55.27 ± 9.17	**0.001**	**59.50**
Gender				
Female	38 (59.38%)	129 (68.25%)		
Male	26 (40.62%)	60 (31.75%)	0.195	
Hypertension	33 (51.56%)	83 (43.92%)	0.289	
Hyperlipidemia	11 (17.19 %)	41 (21.69 %)	0.441	
DM	12 (18.75 %)	13 (6.88%)	**0.008**	
Smoking	16 (25.00 %)	44 (23.28%)	0.780	
Drinking	19 (29.69%)	44 (23.28%)	0.306	
GCS score	9.69 ± 4.23	14.10 ± 1.90	**<0.001**	**13.5**
Surgery				
EVT	18 (28.13%)	62 (32.80%)		
Clipping	46 (71.87%)	127 (67.20%)	0.487	
Hunt-Hess score			**<0.001**	
I	7 (10.94%)	106 (56.08%)		
II	6 (9.38%)	35 (18.52%)	0.106	
III	20 (31.25%)	38 (20.11%)	**<0.001**	
IV	19 (29.69%)	9 (4.76%)	**<0.001**	
V	12 (18.75%)	1 (0.53%)	**<0.001**	
WFNS			**<0.001**	
I	12 (18.75%)	132 (69.84%)		
II	10 (15.63%)	30 (15.87%)	**0.006**	
III	4 (6.25%)	6 (3.17%)	**0.005**	
IV	14 (21.88%)	19 (10.05%)	**<0.001**	
V	24 (37.50%)	2 (1.06%)	**<0.001**	
Aneurysm location				
Anterior circulation	61 (95.31%)	179 (94.71%)	0.850	
Posterior circulation	3 (4.69%)	10 (5.29%)		
mFisher grade			**<0.001**	
I	16 (25%)	119 (62.96%)		
II	14 (21.88%)	30 (15.87%)	**0.003**	
III	13 (20.31%)	33 (17.46%)	**0.011**	
IV	21 (32.81%)	7 (3.70%)	**<0.001**	
ICH	30 (46.88%)	30 (15.87%)	**<0.001**	
IVH	37 (57.81%)	43 (22.75%)	**<0.001**	
BG, mmol/L	9.12 ± 2.72	7.17 ± 1.91	**<0.001**	**7.22**
PLT, *10^9^/L	199.73 ± 70.84	214.02 ± 70.39	0.161	
Hb, g/L	126.94 ± 22.24	127.91 ± 17.03	0.715	
RBC, *10^12^/L	4.73 ± 4.01	4.24 ± 0.49	0.101	
HCT, %	38.34 ± 5.87	38.05 ± 4.45	0.678	
WBC, *10^9^/L	15.82 ± 18.40	10.49 ± 3.78	**<0.001**	**11.92**
Neutrophil, *10^9^/L	11.87 ± 4.58	8.80 ± 3.82	**<0.001**	**9.08**
Lymph, *10^9^/L	0.99 ± 0.56	1.11 ± 0.54	0.107	
Monocyte, *10^9^/L	0.73 ± 0.42	0.54 ± 0.24	**<0.001**	**0.66**
NLR	16.17 ± 10.41	10.27 ± 8.06	**<0.001**	**17.39**
PLR	265.35 ± 204.67	226.10 ± 123.72	0.068	
LMR	1.69 ± 1.12	2.44 ± 1.58	**<0.001**	**1.33**
PWR	16.02 ± 7.88	22.29 ± 9.36	**<0.001**	**15.16**
SIRI	12.58 ± 12.71	5.64 ± 5.81	**<0.001**	**5.52**
RDWCV	13.34 ± 1.95	12.89 ± 1.36	0.052	
RDWSD, fl	43.70 ± 3.91	42.11 ± 3.53	**0.004**	**43.05**

DM, diabetes mellitus; GCS, Glasgow coma scale; EVT, endovascular therapy; WFNS, world federal neurological scale; mFisher grade, modified Fisher grade; ICH, intracranial hemorrhage; IVH, intraventricular hemorrhage; BG, blood glucose; PLT, blood platelet; Hb, hemoglobin; RBC, red blood cell; HCT, hematocrit; WBC, white blood cell; NLR, neutrophil-lymphocyte ratio; PLR, platelet to lymphocyte ratio; LMR, lymphocyte to monocyte ratio; PWR, PLT to WBC ratio, SIRI, systemic inflammation response index; RDW-CV, red blood cell distribution coefficient of variation; RDW-SD, red blood cell distribution width-standard deviation. Variables showing statistical significance (*p* < 0.05) are in bold.

### Statistical analysis

Statistical analysis was performed using SPSS 20.0 (IBM Inc, Chicago, IL). Continuous variables were expressed as mean ± SD. Categorical variables were expressed as frequencies (percentages). The Kolmogorov–Smirnov test was performed to determine whether the parameter dataset was normally distributed. A univariate logistic analysis was used for all variables. Significant parameters were then entered into a multivariate logistic regression using the stepwise forward method to identify the independent risk factors. A logistic model (Model-Logit) was established based on the independent risk factors. We used risk factor categories to develop a new model (Model-Cat). Receiver operating characteristic (ROC) curves were generated to calculate significant variables of areas under the curve (AUCs) and cutoffs. The Delong test was performed to compare the AUCs. Based on the literature (15), predictive scores and corresponding risk estimate were calculated. Differences where *p* < 0.05 were considered statistically significant.

## Results

### Patient demographics

We included 253 patients with aSAH. Of these, 64 patients developed HAP, with an incidence of 25.30%. In the HAP group, 64 patients included 38 females (59.38%) and 26 males (40.62%) with a mean age of 59.59 (range 44–76 years). In the non-HAP group, 189 patients included 129 females and 60 males with a mean age of 55.27 (range 28–77 years). Demographics are displayed in Table 1.

### Predictive factors for HAP

**Figure 2:**
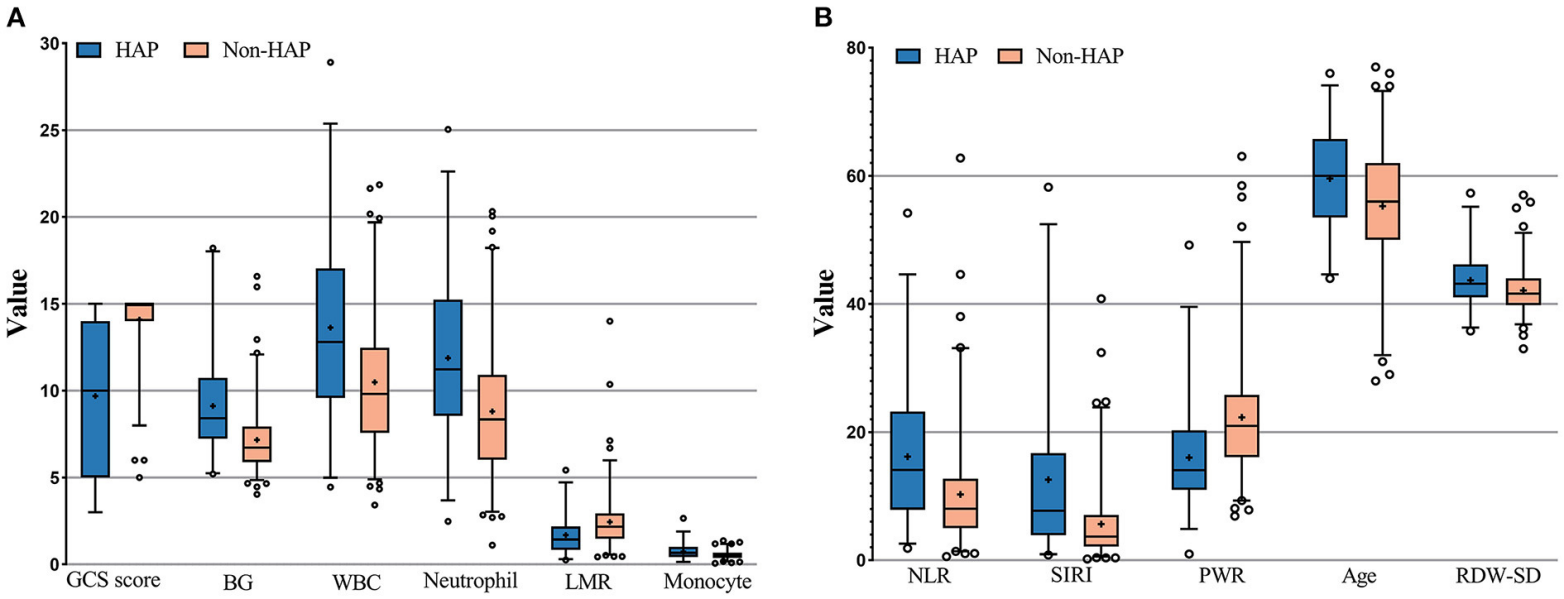
**(A,B)** Measurement of the significant variables in univariate analysis; GCS score, Glasgow coma scale score; LMR, Leukocyte to monocyte ratio; NLF, neutrophil to lymphocyte ratio; SIRI, systemic inflammation response index; PWR, platelet to white blood cell ratio; RDW-SD, red blood cell distribution width standard deviation.

The results of the calculation and univariate analysis are shown in Table 1. The distribution of the significant variables is shown in Figure 2. The univariate logistic regression showed that, compared to the non-HAP group, the HAP group had significantly greater age (59.59 vs. 55.27, *p* = 0.001), BG (9.12 vs. 7.17 mmol/L, *p* < 0.001), WBC (15.82 vs. 10.49, *p* < 0.001), neutrophils (11.87 vs. 8.80, *p* < 0.001), monocytes (0.73 vs. 0.54, *p* < 0.001), NLR (16.17 vs. 10.27, *p* < 0.001), SIRI (12.58 vs. 5.64, *p* < 0.001), and RDW-SD (43.70 vs. 42.11, *p* = 0.004). Diabetes mellitus (*p* < 0.001), Hunt-Hess score ≥ III (*p* < 0.001), intracerebral hemorrhage (*p* < 0.001), and intraventricular hemorrhage (*p* < 0.001) were significantly associated with HAP. Lower GCS scores (9.69 vs. 14.10, *p* < 0.001), lymphocyte-to-monocyte ratios (1.69 vs. 2.44, *p* < 0.001) and platelet-to-WBC ratios (16.02 vs. 22.29, *p* < 0.001) were found in the HAP group. WFNS ≥ II, Hunt-Hess grade ≥ III, and modified Fisher grade ≥2 were significantly associated with HAP. The thresholds to discriminate the higher risk of HAP with cutoff values of the significant continuous variables with the highest sensitivity and specificity are displayed in Table 1.

Using the stepwise forward method, the multivariate logistic regression results are displayed in Table 2. The age [*p* = 0.012, odds ratio (OR) = 1.059, confidence interval (CI) = 1.013–1.107], BG (>7.22 mmol/L; *p* = 0.011, OR = 2.781, CI = 1.263–6.119), RDW-SD (*p* = 0.024, OR = 1.118, CI = 1.015–1.231), and GCS score (*p* < 0.001, OR = 0.710, CI = 0.633–0.798) were independent risk factors. Based on these independent risk factors, we established the Model-Logit and the Model-Cat (Table 3). Corresponding points and risk estimates are displayed in Table 4.

**Table 2:** Results of multivariate logistic regression.

**Variables**	**β value**	***p-*value**	**OR value**	**95% CI**
Age	0.057	0.012	1.059	1.013–1.107
BG (>7.22 mmol/L)	1.023	0.011	2.781	1.263–6.119
RDW-SD, fl	0.111	0.024	1.118	1.015–1.231
GCS	−0.342	<0.001	0.710	0.633–0.798
Constant	−5.467	0.041	0.004	

OR, odds ratio; CI, confidence interval; RDW-SD: the width of RBC distribution; GCS, Glasgow coma score; OR, odds ratio; CI, confidence interval.

**Table 3:** Predictive model using risk factor categories.

**Risk factor**	**Categories**	**Reference value**	**W*_*ij*_*-W_i*REF*_**	***D***	**Points**
Age	20–29	24.5	−20	−1.14	−2
	30–39	34.5	−10	−0.57	−1
	40–49	44.5 = W_1REF_	0	0	0
	50–59	55.5	10	0.57	1
	60–69	65.5	20	1.14	2
	70–79	75.5	30	1.71	3
BG, mmol/L	<7.22	0 = W_2REF_	0	0	0
	≥7.22	1	1	1.02	2
GCS score	3–8	5.5	−8.5	2.91	5
	9–12	10.5	−3.5	1.20	2
	13–15	14 = W_3REF_	0	0	0
RDW-SD, fl	<35	34	−6	−0.67	−1
	≥35; <45	40 = W_4REF_	0	0	0
	≥45; <55	50	10	1.11	2
	≥55	60	20	2.22	4

BG, blood glucose; GCS score, Glasgow coma scale score; RDW-SD, red blood cell distribution width standard deviation; W_ij_, reference value; W_REF_, the basic risk value; D, distance, D = β^*^(W_ij_ -W_iREF_); Points_i_ = D_i_/B.

**Table 4:** Estimate of risk corresponding to total scores.

**Total scores**	**Estimate of risk**	**Total scores**	**Estimate of risk**
−3	0.68%	6	53.53%
−2	1.19%	7	67.07%
−1	2.09%	8	78.27%
0	3.63%	9	86.43%
1	6.25%	10	91.85%
2	10.54%	11	95.22%
3	17.24%	12	97.24%
4	26.92%	13	98.42%
5	39.45%	14	99.10%

### Model-Logit and Model-Cat

The Model-Logit was as follows: Logit(*P*) = −5.467 + 0.057 ^*^ age + 1.023 ^*^ BG (>7.22 mmol/L, yes = 1, no = 0) + 0.111 ^*^ RDW-SD−0.342 ^*^ GCS. This model was accurate but inconvenient for clinical use. Therefore, we established a Model-Cat (Table 3) whose method is similar to Wilson et al. (15). Based on our samples. The reference values (W_*ij*_) are displayed in Table 3. We set the basic risk value (W_i*REF*_) of age, BG (>7.22), GCS, and RDW-SD as 44.5, 0, 14, and 40, respectively. When the parameters exceeded the W_i*REF*_, the greater points represented higher risks. The distance (*D*) was calculated based on the equation: $D=\beta_{i}^{*}\left(W_{ij}-W_{REF}\right)$. We set the constant (*B*) change of each risk factor for each point in the model. We regarded every increase of 10 years of the age as one point, as follows: *B* = 10^*^β_*age*_, Points_*i*_ = *D*_*i*_/*B*. Finally, the risk estimate (*P*) corresponding to the total score was based on the following equation:


$$\begin{array}{l} P=\frac{1}{1+\exp\left(-\sum_{i=0}^{p}\beta_{i}\chi_{i}\right)};\overset{p}{\underset{i=0}{\sum }}\beta_{i}\chi_{i}=\beta_{constant}\\ +\beta_{Age}^{*}W_{1REF}+\beta_{BG}^{*}W_{2REF}+\beta_{GCS}^{*}W_{3REF}+\beta_{4}^{*}W_{4REF}\\ \;+B^{*}Total\;score=0.57^{*}Total\;score-\;3.2785. \end{array}$$

Total scores ranged from −3 to 14 points. The total points and risk estimates are displayed in Table 4.

**Figure 3:**
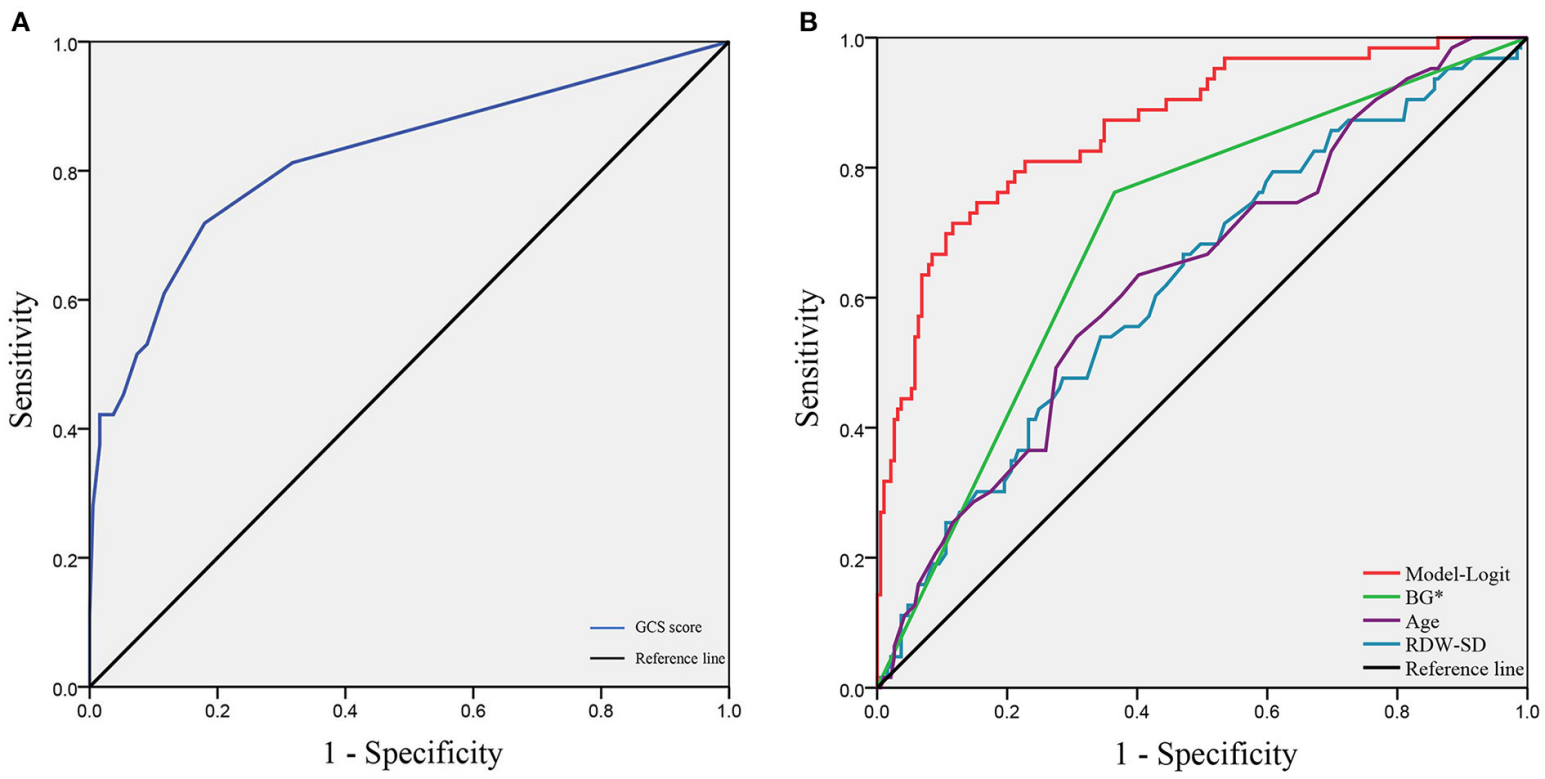
ROC analysis of the Model-Logit and independent risk factors. **(A)** GCS score as a protective role with the AUC of 0.819; **(B)** AUCs of the Model-Logit, BG*, age, and RDW-SD are 0.865, 0.698, 0.634, and 0.625, respectively. Model-Logit, logistic model; GCS score, Glasgow coma scale score; BG*, blood glucose (>7.22 mmol/L); RDW-SD, red blood cell width distribution standard deviation.

**Figure 4:**
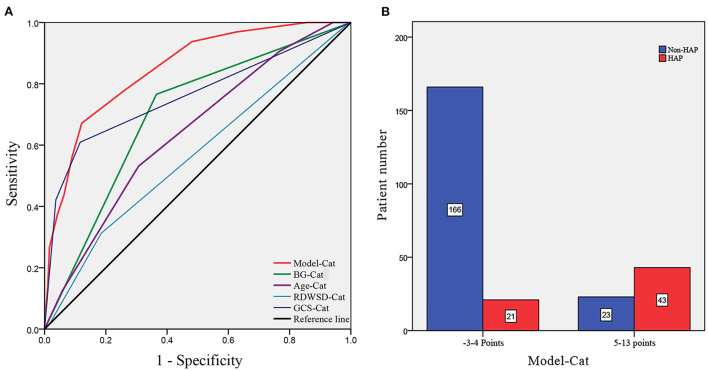
ROC analysis of the Model-Cat and risk level classification. **(A)** AUCs of the Model-Cat, BG-Cat, Age-Cat, RDWSD-Cat, and GCS-Cat were 0.850, 0.700, 0.641, and 0.564, and 0.760, respectively. **(B)** Patient numbers are classified as low-risk level (−3 to 4 points) and high-risk level (5–14 points). Model-Cat, predictive model using risk factor category; Glu-Cat, blood glucose (>7.22 mmol/L) category; Age-Cat, age category; RDWSD-Cat, red blood cell distribution standard deviation category; GCS-Cat, Glasgow coma scale score category.

To verify the model of risk factor categories, we generated ROC curves in both models. In the Model-Logit, the AUC of the Model-Logit, GCS, age, BG (>7.22 mmol/L), and RDW-SD were 0.865, 0.819, 0.634, 0.698, and 0.625, respectively (Figure 3). The Delong test showed that the AUC of the Model-Logit was significantly higher than the GCS (*p* = 0.0386), age (*p* < 0.001), BG (>7.22 mmol/L; *p* < 0.001), and RDW-SD (*p* < 0.001). In the Model-Cat, the AUCs for GCS, age, BG, and RDW-SD were 0.850, 0.760, 0.700, 0.641, and 0.564, respectively (Figure 4A). The Delong test was also performed to compare the ROC curves between the models. The difference in areas was 0.015; however, the AUC of the Model-Logit was insignificantly higher than that of the Model-Cat (*p* = 0.157). This finding suggests that the Model-Cat is convenient, and the accuracy is close to Model-Logit. Cutoff values of the Model-Logit and Model-Cat were −0.849 and 4.5 points, respectively. Therefore, we considered scores of −3 to 4 as the low-risk group and 5–14 as the high-risk group. Low- and high-risk cohorts are represented in Figure 4B.

## Discussion

Aneurysmal subarachnoid hemorrhage (aSAH) accounts for ~5% of strokes (16). Despite substantial advancements in the care of aSAH, the mortality rates are 32%−67%, and one-third become dependent on care (2). About one-third of patients with SAH suffer from systemic infections (mainly pneumonia), which can lead to additional mortality after SAH (3, 5). Cerebral hypoxia due to disturbed cerebral circulation generates exogenous materials that cause early brain injury (2, 17). For these reasons, preventing hypoxia is critical. Pulmonary complications damage air exchange and worsen hypoxia, aggravating brain injury (1, 2). Therefore, evaluating the possibility of developing pneumonia during hospitalizations in the early stage and subsequently avoiding potential risk factors is desirable to prevent HAP and improve outcomes in aSAH patients (4).

Hospital-acquired pneumonia in aSAH requires attention. Kumar et al. (18) demonstrated that individuals with HAP had worse long-term outcomes. Khanzadeh et al. (19) showed NLR could be recommended as a biomarker for predicting infection, particularly pneumonia, in stroke patients. Some studies returned inconsistent results and identified independent risk factors, including weekend admission, Hunt-Hess grade III, external ventricular drain, male sex, use of mannitol, enteral feeding, WFNS score, and neutrophil count (4, 20). They did not apply a specific value to discriminate against high risk. Age, BG, RDW-SD, and GCS were independent risk factors in our study. A Model-Cat was established, and the AUC was close to that of the Model-Logit (0.850 vs. 0.865, Delong test, *p* = 0.157), which could aid practical evaluation.

Advanced age is associated with more risk of HAP in aSAH patients. HAP cohorts had a mean age of 59.59 years old, with a threshold of 59.50 years old. Wang et al. and Ding et al. stressed the significance of age, and their mean value was higher than ours (61, 59.79 vs. 59.50). The optimal threshold was age c60, and the patients with aSAH were at high risk. The World Health Organization defines older people as those over 60. Thus, in clinical use, older people (The optimal threshold w60) with aSAH had a higher risk of HAP.

Blood glucose (>7.22 mmol/L) is a two-category variable applied to the risk threshold with a predictive value. High glucose levels are frequently associated with vasospasm, secondary ischemia, and poor outcomes (2, 21, 22). Hyperglycemia might be expected in aSAH patients due to the transient stress reaction and an acute metabolic response (21). Zhang et al. (23) found that the BG of SAH patients with poor outcomes had a mean value of 7.34 mmol/L, which exceeded the threshold of 7.22 in our study. Eagles et al. (13) identified an optimal BG target in aSAH patients (<9.2 mmol/L), which is close to the BG value (9.12 mmol/L) in our HAP cohort. Abulhasan et al. (24) analyzed 419 patients with aSAH and found that BG (>10 mmol/L) increased the risk of pneumonia. In our study, the risk of HAP would increase 2.781-fold when the admission BG exceeded 7.22 mmol/L.

Low GCS is a common predictor of poor outcomes in intracranial hemorrhage; a pulmonary infection might be the explanation. In our study, the mean value of GCS in the HAP group was lower significantly than in the non-HAP group (9.69 vs. 14.10). Consistent with our results, several studies also found that low GCS was associated with a higher risk of developing pneumonia in aSAH patients (11). Wang et al. (4) found that HAP patients had lower GCS scores than the threshold of GCS in our study (12 < 13.5). Dunn et al. (25) reported an independent association between GCS and dysphagia. Dysphagia could increase the risk of aspiration pneumonia and lung infections. The GCS reflects the extent of the brain injury, and the subsequent impaired cardiopulmonary function and systematic inflammation could produce worse outcomes (4). Chaudhry et al. (3) also stressed that immunodepression is probably the most crucial mechanism leading to pneumonia infections after aSAH. The study of Faura et al. (16) highlighted that stroke generated powerful inflammatory cascades and the peripheral immune system immunosuppression, which could enhance the risk of infection.

Red blood cell distribution width-SD refers to the degree of variation in the volume of red blood corpuscle in circulating plasma (12). Several lines of evidence suggested that RDW-SD was significantly associated with systemic inflammatory responses (26, 27). Nakamura et al. (27) reported that substantial destroyed RBCs follow activated inflammatory responses, which promote hematopoiesis and accelerate the production of immature RBCs. Thus, increased RDW-SD could serve as a proxy for an inflammatory state. An aneurysm rupture would cause brain injury and cause a systematic inflammatory response. Peripheral immunosuppression further aggravates the risk of infection (16). Furthermore, there is no specific value to identify the risk of HAP. In our study, higher RDW-SD could increase the risk of HAP in aSAH patients, with a threshold of 43.05.

Even though any single markers presented good performance in predicting HAP, they were only highlighted by their significance and applied thresholds. Numerous factors contributed to the results. Predictive models could aid these evaluations. Model-Logit was established; it is accurate but inconvenient for clinical use. The Model-Cat using the risk factor categories was superior. The AUC of the Model-Cat was insignificantly lower than that of the Model-Logit (0.850 vs. 0.865, Delong test, *p* = 0.157). Furthermore, the AUCs of models exceeded any single marker. The risk estimate corresponding to the total point could be used in future studies.

Previous studies paid scarce attention to valid predictive models. Wang et al. proposed a model that included simplified scores of WFNS, neutrophils, RBC transfusion, and tracheostomy (4). It performed well with an AUC of 0.808 and a cutoff of 0.2696 to identify high-risk patients. Our Model-Cat presented a higher AUC (0.850 vs. 0.808) and provided risk factor categories to identify individualized risks. The optimal cutoff of the Model-Cat was 4.5, and patients could be divided into low-risk (−3 to 4 points) and high-risk cohorts (5 to 14 points). Effective prevention should be implemented in high-risk HAP patients with aSAH.

There were several limitations in this study. First, we only considered aSAH in a single institute from 2020 to 2022. The limited sample size might have decreased the accuracy of our findings. Second, our study did not consider tracheotomy and mechanical ventilation. We aimed to test the admission state to evaluate the risk of HAP; these factors may cause selection bias. Third, drug usage records were not recorded. Mannitol, crystalloid, nimodipine, anticonvulsant, and proton pump inhibitors might influence the development of HAP. Fourth, some laboratory parameters were not included (e.g., procalcitonin, IL-10, and C-reactive protein). Fifth, we did not involve parameters correlated with the COVID-19, even though the COVID-19 could enhance the possibilities of the aneurysm rupture (28). Finally, some clinical manifestations were not included: dysphagia, dysarthria, and hearing failure (7, 8). In future studies, more cases should be gathered at several centers to identify other independent risk factors and increase the predictive model's accuracy.

## Conclusions

In this retrospective study, age, BG (>7.22 mmol/L), GCS, and RDW-SD were independent risk factors for HAP in aSAH patients. The Model-Cat was close to the Model-Logit but more convenient for practical evaluation. The aSAH patients with total points from 5 to 14 had a high-risk HAP level. They require attention during treatment.

## Data availability statement

The raw data supporting the conclusions of this article will be made available by the authors, without undue reservation.

## Ethics statement

This study was reviewed and approved by institutional ethics committee at Tongji Hospital. Written informed consent was obtained from all participants or their legal guardians/next of kin for their participation in this study.

## Author contributions

S-QH and J-NH: conceptualized the study, wrote the manuscript, collected, and analyzed data. R-DC: revised the draft paper. J-SY: supervision. All authors contributed to the article and approved the submitted version.

## Conflict of interest

The authors declare that the research was conducted in the absence of any commercial or financial relationships that could be construed as a potential conflict of interest.

## Publisher's note

All claims expressed in this article are solely those of the authors and do not necessarily represent those of their affiliated organizations, or those of the publisher, the editors and the reviewers. Any product that may be evaluated in this article, or claim that may be made by its manufacturer, is not guaranteed or endorsed by the publisher.
